# Draft Genome Sequence of the *Paenibacillus* sp. Strain ICGEB2008 (MTCC 5639) Isolated from the Gut of *Helicoverpa armigera*

**DOI:** 10.1128/genomeA.00026-12

**Published:** 2013-01-15

**Authors:** Nidhi Adlakha, Hemant Ritturaj Kushwaha, Raman Rajagopal, Syed Shams Yazdani

**Affiliations:** a Synthetic Biology and Biofuel Group, International Centre for Genetic Engineering and Biotechnology, Aruna Asaf Ali Marg, New Delhi, India; b Department of Zoology, University of Delhi, Delhi, India

## Abstract

*Paenibacillus* sp. strain ICGEB2008 (MTCC 5639) is a Gram-positive cellulolytic bacterium, isolated from the gut of *Helicoverpa armigera*. Here, we report the draft genome sequence of *Paenibacillus* sp. ICGEB2008. The annotation of the ~5.7-Mb sequence indicated a cluster of genes related to the glycosyl hydrolase family and the butanediol biosynthesis pathway.

## GENOME ANNOUNCEMENT

The cellulosic biomass is the most abundant biopolymer on earth and is a promising alternative to fossil fuels. However, the absence of effective enzyme systems for the hydrolysis of cellulosic biomass has become a major hurdle in the process of lignocellulosic biofuel production. Recently, focus has been given towards identifying efficient hydrolytic enzymes from cellulolytic microbes residing in the gut of insects feeding on lignocellulosic biomass. We isolated a novel microbe from the gut of the cotton bollworm, *Helicoverpa armigera*, and characterized its potential to produce various cellulolytic enzymes (1). The strain, named *Paenibacillus* sp. ICGEB2008 (MTCC 5639), is a rod-shape facultative anaerobic bacterium. It produces enzymes for the efficient hydrolysis of lignocellulosic biomass into monomeric fermentable sugars (1, 2) and has an inherent capability to synthesize ethanol and butanediol as major fermentation products (unpublished data); this makes it an attractive host for use in the biofuel production process.

The genome of strain ICGEB2008 was sequenced using an Ion Torrent Personal Genome Machine (PGM) instrument (603.22 Mb; 111-fold coverage). *De novo* assembly using MIRA 3.4.0 (a *de novo* assembler) produced 79 contigs totaling 5,691,612 bp (45.5% G+C content) with an N_50_ of 283,871 bp and the maximum contig size of 910,143 bp. The genome annotation was performed using the RAST server (http://rast.nmpdr.org/) and the output was downloaded in GenBank format (3). Among the predicted 5,153 protein-coding genes, 70% have been assigned putative functions according to the subsystem categorization. A total of 110 tRNA genes encompassing all 20 amino acids were identified using the tRNAscan-SE program (4).

The complete gene clusters coding for cellulolytic genes were predicted from the genome sequence. The genes for cellulose degradation, such as endocellulase and endoxylanase, were present in the contigs that were placed far apart in the genome, but the disaccharidases and specific transporters of such disaccharides were found to be present in the same operon. Few glycosyl hydrolases were predicted to have multiple functional domains for cellulase, xylanase, lichenase, or mannanase activities.

Genes involved in the syntheses of ethanol, 2,3-butanediol, and several other important extracellular metabolites were also identified. 2,3-Butanediol is a precursor for a variety of chemical feedstocks and liquid fuels. The genome sequencing data helped in predicting the pathway involved in the microbial production of butanediol. The genes involved in the 2,3-butanediol pathway coding for alpha-acetolactate decarboxylase, alpha-acetolactate synthase, and butanediol dehydrogenase were shown to be located in two operons, in contrast to a previous report for *Klebsiella* and *Enterobacter*, where all the genes were found to be present in a single operon (5).

A whole-genome comparison with the two completely sequenced *Paenibacillus polymyxa* genomes revealed the close relatedness between the *P. polymyxa* M1 and SC2 strains. The genome information provided here will allow for the genetic manipulation of *Paenibacillus* sp. ICGEB2008 for enhanced biofuel and chemical productions.

### Nucleotide sequence accession numbers. 

This Whole Genome Shotgun project has been deposited at DDBJ/EMBL/GenBank under the accession no. AMQU00000000. The version described in this article is the first version, accession no. AMQU01000000.
